# Multidisciplinary Diagnostic Algorithm for Evaluation of Patients Presenting with a Prosthetic Problem in the Hip or Knee: A Prospective Study

**DOI:** 10.3390/diagnostics10020098

**Published:** 2020-02-11

**Authors:** Vesal Khalid, Henrik Carl Schønheyder, Lone Heimann Larsen, Poul Torben Nielsen, Andreas Kappel, Trine Rolighed Thomsen, Ramune Aleksyniene, Jan Lorenzen, Iben Ørsted, Ole Simonsen, Peter Lüttge Jordal, Sten Rasmussen

**Affiliations:** 1 Orthopaedic Research Unit, Aalborg University Hospital, 9000 Aalborg, Denmarkandreas.kappel@rn.dk (A.K.); ohs@rn.dk (O.S.); sten.rasmussen@rn.dk (S.R.); 2 Department of Orthopaedic Surgery, Aalborg University Hospital, 9000 Aalborg, Denmark; 3 Department of Clinical Medicine, Aalborg University, 9000 Aalborg, Denmark; hcs@rn.dk; 4 Department of Clinical Microbiology, Aalborg University Hospital, 9000 Aalborg, Denmark; lone_heimann@hotmail.com; 5 Center for Microbial Communities, Department of Biotechnology, Chemistry and Environmental Engineering, Aalborg University, 9000 Aalborg, Denmark; trt@teknologisk.dk; 6 Danish Technological Institute, Medical Biotechnology, 8000 Aarhus C, Denmark; jnl@teknologisk.dk (J.L.); plj@teknologisk.dk (P.L.J.); 7 Department of Nuclear Medicine, Aalborg University Hospital, 9000 Aalborg, Denmark; raa@rn.dk; 8 Department of Infectious Disease, Aalborg University Hospital, 9000 Aalborg, Denmark; iben.oersted@rn.dk

**Keywords:** prosthesis, infection, algorithm, diagnosis

## Abstract

The predominant indications for revision surgery after total hip (THA) or knee arthroplasty (TKA) are an aseptic failure (AF) and prosthetic joint infection (PJI). Accurate diagnosis is crucial. Therefore, we evaluated prospectively a multidisciplinary diagnostic algorithm including multi-modal radionucleid imaging (RNI) and extended microbiological diagnostics. If the surgeon suspected PJI or AF, revision surgery was performed with multiple samples obtained in parallel for special culture procedures and later molecular analyses. Alternatively, if the underlying cause was not evident, RNI was scheduled comprising ^99^Tc—HDP SPECT/CT, ^111^In-labeled white blood cells combined with ^99^Tc-nanocoll bone marrow SPECT/CT, and ^18^F-FDG PET/CT. A multidisciplinary clinical team made a recommendation on the indication for a diagnostic procedure guided by RNI images or revision surgery. A total of 156 patients with 163 arthroplasties were included. Fifty-five patients underwent RNI. In all, 118 revision surgeries were performed in 112 patients: 71 on the indication of AF and 41 revision of PJI. Thirty-four patients were concluded with chronic pain, and revision surgery refrained. The effective median follow-up period was 13 months. A structured approach offered by the algorithm was useful for the clinician in the evaluation of patients with a failing TKA or THA. Surgical revision was possibly obviated in approximately 20% of patients where an explanation or cause of failure was not found. The algorithm served as an effective tool.

## 1. Introduction

Complications after total hip (THA) or knee arthroplasties (TKA) present an increasing challenge for the health services, primarily reflecting a rising number of primary surgeries [1]. In general, complications can be divided into a prosthetic joint infection (PJI), aseptic failure (AF) (most of which are implant loosening, instability, and polyethylene wear) [2], and an exclusion diagnosis of chronic pain. In Scandinavian countries, the leading indication for revision surgery is AF, followed by PJI [3,4,5]. The risk of a PJI is reported to be one to two percent and is associated with appreciable morbidity, and are complex to diagnose and treat [6,7,8]. Previous studies have projected a threefold rise in the number of revision surgeries due to PJI by the year 2030 [9].

Appropriate diagnosis is paramount for successful treatment. Pain, not being a discriminating symptom, is the most frequent clinical manifestation in PJI and AF [10]. Pre- and intraoperative diagnosis of acute infection is often straightforward, but overt local or systemic findings can be lacking, hence being dependent on a microbiological diagnosis. Cases with culture-negative infections pose a special challenge [11], and a causative role of low-grade infection in AF has been indicated in a number of studies [12,13,14,15]. Finally, non-confirmation of a preoperative diagnosis of chronic infection by culture may leave a notion that the procedure was superfluous.

Efforts have been made to reach a consensus regarding diagnosing a prosthetic problem [16]. However, an international consensus has not been achieved, and surgeons must decide individually which diagnostic tools to employ [17]. Detailed patient history and meticulous clinical examination is fundamental and can be supplemented in a number of ways [18].

Culture methods have been the main diagnostic tool for PJI [11,18,19]. An intraoperative sampling of multiple biopsies became a standard in the 1990s, and more recently, laboratory processing of prosthetic components has become an adjunct to detect biofilm infections. Gene amplification methods have been introduced in various forms and evaluated in a number of studies [20,21].

A prospective study has not yet been performed with a diagnostic algorithm incorporating RNI, optimized sampling logistics, culturing methods, and 16S *rRNA* gene polymerase chain reaction (PCR), and amplicon sequencing. Our hypothesis is that a structured multidisciplinary algorithm is applicable in a clinical setting and can improve diagnosis in patients experiencing a post-hip or knee replacement problem.

## 2. Materials and Methods

**Figure 1:**
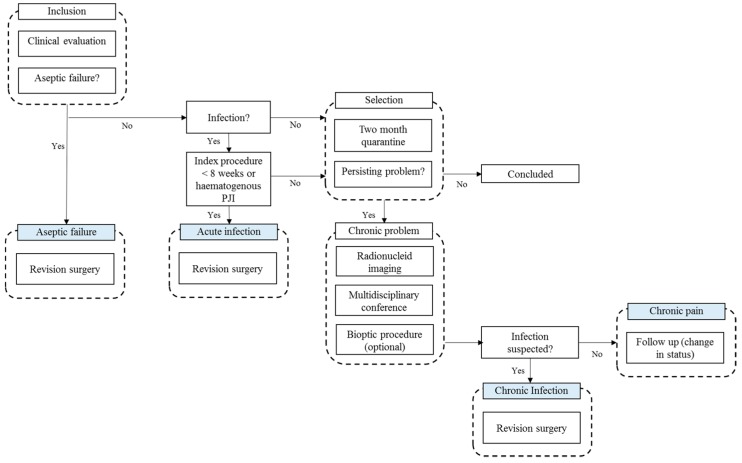
The algorithm.

This study was conducted in North Denmark Region from December 2011 to January 2014 within the framework of an innovation consortium with participation of clinical departments, universities, industry, and the Danish Technology Institute (Danish acronym PRIS). Department of Orthopedic Surgery, Aalborg University Hospital was responsible for inclusion, treatment, and coordination. A multidisciplinary algorithm aimed to improve the diagnosis of patients presenting with a prosthetic problem related to either a TKA or THA (Figure 1). The main difference from the standard course for such patients was the option for RNI and extended set of samples obtained during revision surgery.

### 2.1. Inclusion

Patients with TKA or THA were referred by either general practitioners or other clinical departments. Inclusion criteria were a prosthetic failure and suspected infection. Failure was defined as pain and a mechanical problem (loosening or wear evident by X-ray imaging and physical examination). Exclusion criteria were recurrent dislocation of the hip arthroplasty or age below 18 years.

Clinical evaluation was performed by a specialized team of orthopedic surgeons. Standard biochemical tests and X-ray imaging were requested if necessary but was not an inclusion criterion.

The overall clinical assessment at inclusion was the outset for the algorithm. Surgery for acute infection was scheduled if PJI was suspected clinically within eight weeks from the index procedure, or a hematogenous infection was suspected in a septic patient. If PJI was suspected after 8 weeks since the index procedure, an evaluation for a chronic problem was scheduled. If there was no clinical suspicion of PJI, patients were quarantined for eight weeks after which they were concluded if symptoms remitted. If not, evaluation for a chronic problem continued. Revision for AF was scheduled if radiographic or clinical signs of failure were present, and PJI was not suspected.

### 2.2. Evaluation of a Chronic Problem

A chronic problem was suspected if none of the criteria above were satisfied. RNI was performed on three consecutive days, including ^99^Tc—HDP SPECT/CT, ^111^In-labeled white blood cell (WBC) combined with ^99^Tc-nanocol bone marrow SPECT/CT, and ^18^F-FDG PET/CT, see Appendix A for RNI-protocol. In a multidisciplinary conference, specialists in nuclear imaging, orthopedic surgery, and clinical microbiology evaluated the findings. If findings were compatible with infection, a diagnostic procedure or revision surgery was recommended. In the absence of findings suggestive of PJI, patients were diagnosed as having a chronic pain problem, and follow-up for change in status was planned.

### 2.3. Revision Surgery

Sampling during revision surgery was identical regardless of indication. Before the administration of antibiotics, five periprosthetic synovial tissue biopsies were obtained, according to Kamme & Lindberg [22], as is the routine in the Department of Orthopedic Surgery. Intraoperative project samples followed a previously published protocol [23]. Project samples included triplicates of joint fluid, periprosthetic synovial tissue and bone biopsies, and swabs from the surface of the prosthesis. This sampling strategy made it possible to evaluate experimental methods. Any removed prosthetic components were handled aseptically and subjected to sonication according to a previously published protocol [24]. For more details, see Appendix B. Protocol samples were subject to bacteriological culturing for 14 days, 16S *rRNA* gene PCR followed by amplicon sequencing, and fluorescence in situ hybridization (FISH) (optional). Surgical treatment was not the scope of this study and was left to the surgeon’s discretion.

### 2.4. Clinical Follow-Up

Clinical follow-up was by appointment with the surgeon within six months. A unique civil registration number is used for medical records and laboratory information systems. In August 2015, the first author made a review of later contacts with the Department of Orthopedics and any microbiological samples of relevance (wwLab, Autonik, Sweden).

### 2.5. Data Sources

Baseline characteristics of patients, comorbidities, previous history of the affected joint, and prior antibiotic treatment were obtained from medical records, and blood biochemistry values were from the laboratory information system (Labka, CSC, Denmark) (C-reactive protein (CRP) normal range ≤8 mg/L; WBC count normal range 3.5–10.0 × 10^9^/L).

### 2.6. Diagnosis

Following the algorithm, patients obtained either a diagnosis of acute infection, chronic infection, AF, or chronic pain. Acute infection was a clinical diagnosis, and joint aspiration was recommended. Diagnosis of AF and chronic infection were supported by clinical findings and RNI, joint aspiration, and bioptic procedures being optional. Chronic pain was a diagnosis reached by the exclusion of the aforementioned diagnoses (i.e., clinical suspicion not supporting AF or PJI and absence of ‘hot-spots’ by RNI and a bioptic procedure with a negative culture).

**Figure 2:**
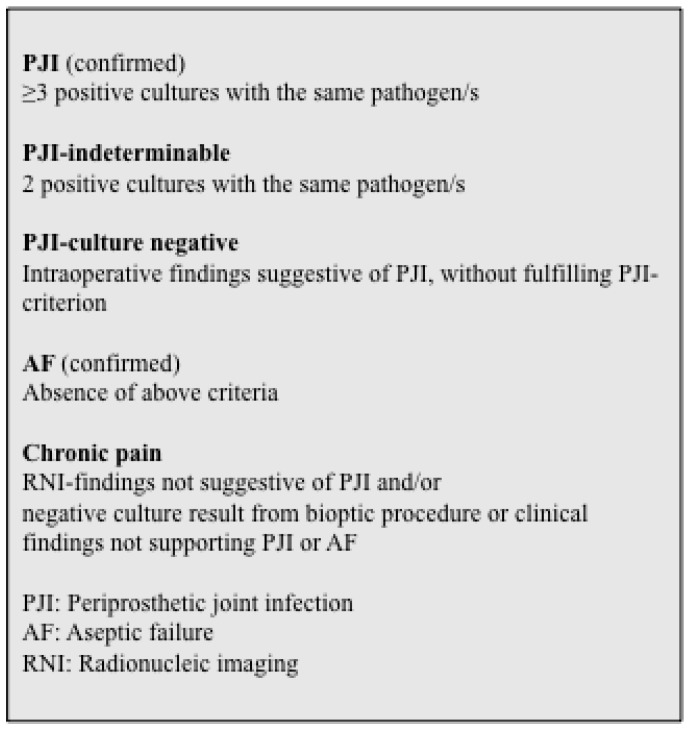
Definition of postoperative and post-radionucleid diagnosis.

In patients undergoing revision surgery, confirmation of PJI required positive culture reports for at least three of five periprosthetic soft tissue biopsies with an identical microorganism(s). This criterion had been used by the Department of Orthopedic Surgery since the 1990s, and a validation study has previously been performed for patients with knee arthroplasty [22,25]. Less stringent criteria have been proposed by others [26], and therefore, an additional analysis was performed for patients with two positive tissue biopsy cultures with identical microorganism(s). A diagnosis of culture-negative PJI was made if the clinical findings, including intra-operative view, was suggestive of PJI without fulfilling other criteria for PJI. Definitions of postoperative and post RNI diagnoses are shown in Figure 2.

16S *RNA* gene PCR and amplicon sequencing was performed after closing the study, and thus, the results were not available to the surgeon.

### 2.7. Case Definition and Data Analysis

A case was defined by a specific arthroplasty and a 6-month follow-up period from study entry. After that period inclusion of the same arthroplasty was permissible as a new case. This arbitrary time limit was chosen for defining a new primary infection. Revision prompted by possible recurrence within six months and a debut of symptoms from another arthroplasty were likewise included as separate cases regardless of the timespan from the previous inclusion.

For the primary analysis, cases were divided into four groups according to the algorithm: acute infection, chronic infection, AF, and chronic pain. Next, PJI and AF cases were stratified based on culture reports from revision surgery: PJI or AF cases could be confirmed or rejected, and as a consequence being referred to as the other diagnostic group. Cases for whom the modified criterion for PJI was fulfilled (i.e., only two culture-positive periprosthetic tissue biopsies) were designated ‘PJI indeterminate’. If the diagnosis of PJI was supported by intraoperative observations but not by conclusive positive cultures, the case was referred to as ‘PJI culture-negative’.

Patients evaluated with RNI were assessed post hoc by a multidisciplinary team (MDT) blinded to patient identity and imaging results. These 55 patients were divided into three groups: PJI, no PJI, and indeterminable (see Appendix C for further description of the MDT-process).

Comparisons were made by Fisher exact test, and binomial confidence limits were calculated (Stata 12, Stata Corp., Texas).

Approval of the PRIS project was obtained from the Research Ethics Committee for the North Denmark Region (N-20110022) and the Danish Data Protection Agency (2008-58-0028).

## 3. Results

**Figure 3:**
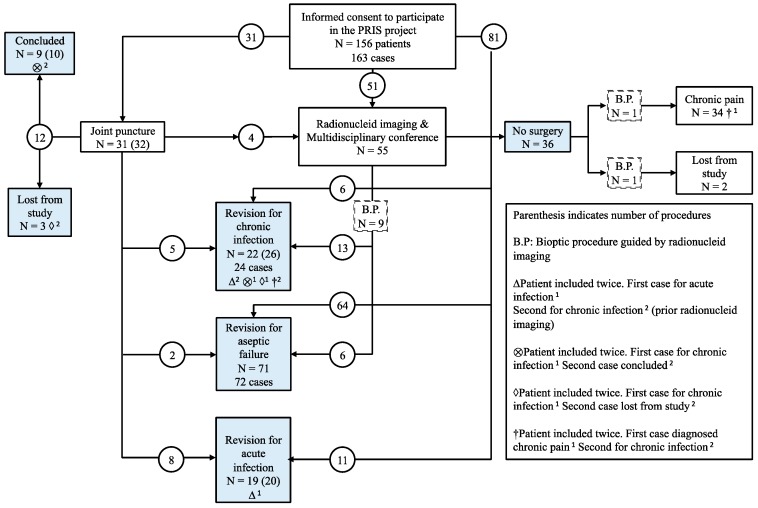
Flowchart.

A total of 156 patients (163 cases) were included (85 TKA and 71 THA). A total of 118 revision surgeries were performed in 112 patients. The patient flow is depicted in Figure 3. Descriptive characteristics of patients at baseline, during the study and follow-up, are shown in Table 1. Key pre- and postoperative findings from the study are shown in Table 2.

We applied logistic regression models to patients with PJI (acute and chronic), AF or chronic pain to define the strength of the association with nine variables (sex, age, Body Mass Index (BMI), C-Reactive Protein (CRP), White Blood Cell (WBC), TKA or THA, prosthesis age in year and number of comorbidities) as the independent variables.

Suspected and confirmed AF was negatively associated to elevated CRP (*p* = 0.001 and *p* = 0.01)).

Acute infection was associated with increased CRP (*p* = 0.002). Chronic infection was associated with male sex (*p* = 0.030), high BMI (*p* = 0.038) and number of comorbidities (*p* = 0.011).

PJI and Indeterminable were associated with increased CRP (*p* = 0.004 and *p* = 0.042). Chronic pain as an outcome was associated with increased BMI (*p* = 0.019).

The negative predictive value of CRP < 7.0 and no infection was 100% in cases of AF (91.0–100, 95% CI) and 100% in cases of chronic pain (80.5–100, 95% CI). However, the sample size was small, with few events.

### 3.1. Aseptic Failure

71 patients (72 cases) underwent revision in the AF group. In two patients, a joint aspirate was culture-negative, and six patients underwent RNI not conclusive for PJI or AF. Five patients (7.0%) were diagnosed postoperatively as PJI and treated at the surgeon’s discretion; two patients remained ‘PJI indeterminable’. During follow-up, one patient was readmitted beyond the study and treated for PJI. Findings from the second revision did not suggest a causal relation with the condition prior to the first revision.

### 3.2. Acute Infection

Nineteen patients underwent 20 revisions (one patient had two revisions of the same joint within six months), and eight patients had prior joint aspiration. PJI was confirmed in 16 patients (17 revisions); one patient remained ‘PJI indeterminable’ and two ‘PJI culture negative’.

### 3.3. Chronic Problem

A total of 55 patients were evaluated for a chronic problem, 19 patients of whom underwent revision surgery (13 for PJI and six for AF, respectively). AF was not suspected at inclusion in the six patients. RNI was not conclusive for AF or PJI, although clinical judgment supported revision surgery for AF in these six patients.

One patient had been treated within the study seven months earlier for an acute infection of the same joint. Following RNI, revision for chronic infection was scheduled. Prior to imaging, four had a culture-negative joint aspiration within the study. A bioptic procedure was done in 11 patients guided by results from RNI (seven percutaneous biopsies and four joint aspirates).

Two patients strongly suspected of PJI were lost from the study. One patient had a bioptic procedure with positive culture suggesting PJI and was not fit for surgery. Another patient had revision surgery beyond the study for PJI.

Chronic pain was concluded in 34 patients. Three patients had a culture-negative bioptic procedure guided by RNI. One of these patients was included again 11 months later and underwent revision surgery for chronic infection. Another two patients underwent revision surgery for AF during follow-up; standard cultures were negative. Results from RNI will be published elsewhere.

### 3.4. Chronic Infection

Twenty-six revision surgeries (two patients with revision of the same joint within six months) were performed in 22 patients (24 cases; one patient was included nine months later with the same joint, and one with TKA and THA was included with both four months apart). Thirteen of these patients had undergone RNI, as described above.

PJI was confirmed in 17 patients (20 revisions). ‘PJI indeterminable’ applied to three patients (four revisions). In one patient, PJI was not confirmed and thus diagnosed as AF. PJI-culture negative was concluded in one patient.

Nine patients had a culture-negative joint aspiration and were concluded without revision surgery. Three patients were lost from the study after joint aspiration, one being treated for AF and two for PJI outside the study.

## 4. Discussion

In this study, we summarize major findings in 156 prospectively recruited patients representing 163 cases who were assessed by the use of a multidisciplinary diagnostic algorithm. To our knowledge, this is the first study of its kind. The few exclusion criteria make the study representative for patients with a problem related to a THA or TKA.

It is important to distinguish between PJI and AF, as the appropriate course of treatment is different. A definite diagnosis remains a challenge, especially when surgery is not planned. Patients undergoing revision surgery are at eight times higher risk of a subsequent PJI compared with patients undergoing a primary arthroplasty [24]. Therefore, alternative methods are warranted in order to strengthen indications for revision surgery.

Joint aspiration was discouraged in the initial evaluation due to possible interference with RNI [27]. Following RNI, a recommendation was made for a diagnostic procedure or revision surgery. Invasive diagnostic procedures are a valuable option and should be employed when relevant. Despite using sterile techniques, concern exists for introducing infection in a sterile joint when employing invasive diagnostic procedures. Therefore, the decision-making process after evaluation of the chronic problem was left to the discretion of the surgeon and the patient. Hence, final diagnoses after RNI were made either by tissue culture (from a bioptic procedure or revision surgery) or a clinical follow-up, as reported by Aksoy et al. [25]. Details from RNI will be published elsewhere.

Elevated CRP was associated with PJI and normal CRP with confirmed AF. These findings have been consistent with other studies [17,28].

Sensitization in the peripheral and central nervous system has been demonstrated in patients with chronic pain after TKA [29]. “Primum non nocere” is advised in these cases as the underlying cause may not require surgical treatment. Following the algorithm, we diagnosed 34 patients with chronic pain, one and two of whom were diagnosed during follow-up with PJI and AF, respectively. Consequently, 3/34 of patients diagnosed with chronic pain were misdiagnosed. The remaining 31 patients did not undergo revision. The group had a clinical follow-up of 12 months (mean) and at the time of writing, none of these patients had a diagnosis of PJI recorded in national databases during 27 months of follow-up (mean). We interpret this to support the study diagnosis. Patients presenting with a failing THA or TKA, without clinical findings supporting the symptoms of pain pose a challenge. Special attention should be given to these patients before offering an invasive procedure.

Diagnostic algorithms exist for evaluating failing arthroplasties in other joints, such as in shoulders and ankles [30,31]. Although similarities in indication for revision surgery (PJI and AF), this algorithm does not apply for evaluating other arthroplasties. It is noteworthy that principles for diagnosis are similar; however, differences also exist, e.g., distribution of microorganisms [30]. It would; however, be obvious to test this algorithm in other failing arthroplasties.

Recently much attention has been given to alpha-defensin tested in synovial fluid in failing TKA and THA [19]. Alpha-defensin was not a part of the algorithm in this study. However, it would have an interesting and relevant supplement in the analysis of joint fluid for comparison to the protocol samples.

Different sets of diagnostic criteria for PJI have been proposed by international societies and individual authors including the European Society of Clinical Microbiology and Infectious Diseases [32], the Musculoskeletal Infection Society [33], the Infectious Disease Society of America [34], and the group of Zimmerli et al. [35]. We chose in 2011 to maintain conservative criteria for PJI and allowing the project samples and extended laboratory workup being a foundation for revised criteria. Our post hoc analysis using conservative criteria confirmed the clinical diagnoses in 90 patients (93 cases). Six patients changed diagnosis, and six additional patients fulfilled a modified criterion for PJI and are reported here as ‘PJI indeterminable’. We foresee that results obtained with 16S *rRNA* gene PCR followed by amplicon sequencing may help to clarify this latter group. Data from culture and 16S rRNA in the different specimen types are published elsewhere [36].

The strength of this study was the prospective design, the added value from the multidisciplinary cooperation, and the implementation of a new diagnostic strategy offered through the algorithm. The validity of the study was increased by the diverse history of patients, and the recognition of patients’ and surgeons’ autonomy. Still, we did not foresee the complex trajectories of patients within the study (Figure 3).

Limitations of the study are notable: RNI has not yet become standard techniques for TKA- and THA-related problems, and the interpretation had to be made cautiously. Therefore, it was unfortunate that bioptic procedures were not feasible for a sizable part of the patients. The evaluation of all patients undergoing RNI by an MDT was meant to compensate for this information bias, and it can be an effective tool in similar prospective studies. We did not measure applicability or improvement directly. The clinicians found that the algorithm qualified the clinical choice of treatment using the stepwise approach. RNI was not planned as a diagnostic tool for AF. Nevertheless, six patients were treated with revision surgery for AF after RNI.

Patient flow in this study was highly individualized and reflected, in part, the complexity of this patient group. The a priori diagnosis may be changed through the clinical course based on laboratory results and clinical findings.

The risk of PJI increases with the number of revisions, and we were reluctant to exclude patients at elevated risk. Still, the study would have benefitted from a less pragmatic case definition.

In order to maintain consistency in diagnosis and treatment, we chose not to revise our routine diagnostic criteria for PJI with at least three positive cultures with the same pathogen. However, we foresee that the criterion of at least three concordant culture reports for sets of periprosthetic tissue biopsies can be decreased to two or more culture-positive biopsies (patients fulfilling a relaxed criterion only are here reported as ‘PJI indeterminable’).

## 5. Conclusions

Surgical revision was possibly obviated in approximately 20% of patients presenting with a chronic problem where an explanation or cause of failure was not found. However, clinical judgment remained as a key element in the algorithm.

Accurate and efficient diagnostics are imperative for correct diagnosis and treatment. Assessment of a failed THA and TKA should follow a strict workup. Applying a multidisciplinary diagnostic algorithm in a clinical setting is feasible. It is our recommendation that a structured approach with an algorithm serves as a useful tool in optimizing diagnostics and resulting in personalized patient treatment. The comprehensive investigation in our algorithm is not recommended in routine cases but may serve as an option in difficult cases.

## Figures and Tables

**Table 1:** Characteristics of 156 patients experiencing a post-hip or knee replacement problem.

Characteristics	Values
Age, years (median, IQR)	68.4 (63.0–76.0)
Sex, number (females)	80
Joint prosthesis (*n*, %)	
Hip	71 (45.5%)
Knee	85 (54.5%)
Comorbidities (*n*, %)	
Rheumatic disease	18 (11.5%)
Cardiovascular disease	30 (19.2%)
Diabetes mellitus	23 (14.7%)
Cancer	16 (10.3%)
COPD	5 (3.2%)
Biological immunotherapy	17 (10.9%)
Prosthesis, age, years (median, IQR)	8.1 (1.6–14.4)
Indication for previous surgery (*n*, %)	
Aseptic failure	23 (14.7%)
Prosthetic joint infection	26 (16.7%)
Prior antibiotic treatment (*n*, %))	30 (19.2%)
Algorithm (*n*, %)	
Initial joint puncture	31 (19.9%)
Radionucleid imaging	55 (35.3%)
Bioptic procedure in patients with radionucleid imaging	11 (20%)
Assesment by multidisciplinary team (MDT) (*n*, %)	55 (35.3%)
Loss to follow-up including death (*n*, %)	5 (%)
Follow-up period, days (median, range)	398 (3–1244)

**Table 2:** Key pre- and postoperative findings.

Key Findings				
Preliminary diagnosis. No. of patients (and revisions)	Aseptic failure *n* = 71 (72)	Acute infection *n* = 19 * (20)	Chronic infection *n* = 22 ^∆,^* (26)	Chronic pain *n* = 34
Age, years (mean, SD)	70.0 (12.5)	72.5 (10.8)	63.5 (11.8)	66.0 (10.2)
Sex, number (females)	40	13	6	16
Joint				
Hip	35	7	11	14
Knee	36	12	11	20
Prosthesis, age, years (median, interquartile range (0.25–0.75)	9.3 (2.8–15.7)	7.0 (0.5–14.7)	10.0 (1.5–15.0)	9.3 (1.6–9.4)
Blood biochemistry values and antibiotic treatment (within 4 weeks) at inclusion				
CRP (µg/mL, median and range)	8 (1–92)	164 (11–394)	47 (5–345)	6 (0–19)
WBC (range 10^9^/L, median and range)	7 (3–12)	10 (5–24)	7 (4–11)	14 (2–15)
Prior antibiotic treatment (no.)	3	11	8	2
Confirmed diagnosis based on work up of project samples from revision surgery				
Aseptic failure (confirmed)	64 (65)	0	1	-
Prosthetic joint infection (confirmed)	5	16 (17)	17 (20)	-
PJI-culture negative	0	2	1	-
PJI-indeterminable	2	1	3 (4)	-
Follow-up				
Follow-up period, days (median and range)	391 (3–1131)	292 (15–1065)	498 (21–1031)	379 (14–1095)
Indication for revision during follow-up (*n*)				
Aseptic failure	4	1	5	2
Prosthetic joint infection	1	4	4	1 ^∆^

^∆^ One patient included twice. Firstly, diagnosed with chronic pain after radionucleid imaging. Secondly, with chronic infection in the study during follow-up. * One patient included twice. Firstly, revision for acute infection. Secondly for chronic infection (prior radionucleid imaging).

## Appendix A

Protocols for radionuclide imaging procedures in PRIS-project

The nuclear imaging procedures performed in the Department of Nuclear Imaging in Aalborg University Hospital within one week for every patient evaluated for chronic problems on three consecutive days, followed by a multidisciplinary team (MDT) meeting for further decision-making.

All the imaging modalities were performed over the knee or hip region respectfully.

Day 1

Bone scan with ^99^Tc—HDP performed as static uptake followed by SPECT/CT with low dose CT on Siemens Hybrid scanner (Symbia T16, Siemens Medical Solutions, Erlangen, Germany) in accordance with institutional procedures.

- The mean injected activity of ^99^Tc—HDP was 750 MBq.
- The bone scan acquired approximately 2–3 h after tracer injection.
- Uptake parameters: static uptake 10 min, matrix size 256c × 256, zoom factor 1.00. SPET/CT 20 sec/view, 32 views, matrix size 128 × 128, zoom factor 1.00, iterative reconstruction.

Day 2

White blood cell (WBC) labeling procedure with ^111^In. Static uptake over the lungs 30 min after reinjection due to quality control of labeling.

^18^F FDG PET/CT on GE Discovery VCT PET/CT scanner (GE Healthcare, Waukesha, WI, USA) in accordance with institutional procedures.

- The mean injected activity of ^18^F FDG was 370 MBq.
- PET/CT was acquired approximately 60 min after tracer injection.

Day 3

^111^In-labeled WBC static and SPECT/CT uptake with low dose CT on Siemens Hybrid scanner (Symbia T16, Siemens Medical Solutions, Erlangen, Germany).

Simultaneous ^111^In-labeled WBC scan combined with ^99^Tc-nanocoll bone marrow scan with SPECT/CT low dose CT, so-called DUAL scan performed on Siemens Hybrid scanner (Symbia T16, Siemens Medical Solutions, Erlangen, Germany).

- The mean injected ^111^In-labeled WBC activity was 20 MBq.
- The mean injected activity for ^99^Tc-nanocoll was 500 MBq.
- Simultaneous Dual uptake performed 24 h after ^111^In-labeled WBC reinjection and 1 h after ^99^Tc-nanocoll injection.
- Uptake parameters: static uptake 15 min, matrix size 256 × 256, zoom factor 1.00. SPET/CT 45 sec/view, 32 views, matrix size 128 × 128, zoom factor 1.00, iterative reconstruction.

## Appendix B

Protocol for samples obtained during revision surgery:

Project samples: PS

Standard tissue samples: STS

In order to minimize contamination, joint fluid was aspirated once the joint was exposed, but prior to incision of the capsule (PS). Once the capsule was incised, three swabs were taken from the surface of the implant, in THA from the femoral head and in TKA from the femoral component. Immediately afterward, three synovial biopsies were taken from the vicinity of the prosthesis (PS). Next in line were five synovial tissue biopsies (STS) taken from the same area as the three samples. If only the polyethylene insert was exchanged, three periprosthetic bone biopsies were taken with a trocar from the bone-joint interface (PS). If other components were removed, bone sampling was withheld and taken from the exposed bone surface. All removed components were collected and placed directly in an assigned container by the surgeon (PS).

Sampling was done with sterile disposable utensils separate for each sample. Antibiotics were withheld until all samples were collected. Routine antibiotic treatment was intravenous cefuroxime until culture results were available (negative results were informed on day 6).

## Appendix C

Decision-making based on radionucleid imaging was left to the discretion of the clinician based on the recommendation from the multidisciplinary conference. For patients who did not undergo a bioptic procedure or revision surgery, laboratory results for diagnostic samples were lacking. To fill this void, a multidisciplinary team (MDT) was appointed to assist in classifying these patients after the study had been closed. The team consisted of three consultants who were specialists in infectious disease, clinical microbiology, and orthopedic surgery, respectively. A comprehensive summary of all available medical records was prepared for each patient spanning the period from the primary arthroplasty to the study end. The main items in the data extract are shown in Table 1.

The team was blinded from each other, and descriptions of nuclear imaging performed as part of the PRIS-project. Each member was asked to conclude:

PJI

PJI effectively ruled out

If a conclusion was not possible, the members were presented with three scenarios.

Scenario A; Nuclear imaging supports suspicion of PJI

Scenario B: Nuclear imaging does not support the suspicion of PJI

Scenario C: Nuclear imaging is inconclusive

For each scenario, members were asked to decide if the additional information was sufficient to conclude 1 or 2 (as above). Conclusions were collected, and agreement defined as two of three identical conclusions.

**Table A1:** Main items in the data extraction.

Baseline Data	
Arthroplasty	Indication for primary arthroplasty All other implants including other arthroplasties Revision surgery of any other implants including treatment and microbiological results
Comorbidities	Inflammatory disease, diabetes (type 1 and 2), cardiovascular disease, pulmonary disease, cancer, biological immunotherapy
Biochemistry	CRP, erythrocyte sedimentation rate (ESR), white blood cell count (WBC)
Clinicians’ evaluation	A complete extract was made from the Department of Orthopedic Surgery for the joint in question, including details from clinical examination (pain, local signs of infection)
Imaging prior to PRIS-study, ‘Prosthetic-Related Infection and Pain’ (Danish acronym)	Extracts of all available descriptions including plain radiographs, ultrasound, magnetic resonance (MR) scan, X-ray computed tomography (CT), radionucleid imaging (beyond PRIS)
Study period and follow-up	
Clinicians evaluation	Extracts of records from the Department of Orthopedic Surgery, including clinical evaluation (pain, local signs of infection) and invasive procedures (joint aspiration, revision surgery, surgeon’s intraoperative view). Material regarding PRIS-radionucleid imaging was omitted
Biochemistry	as above
Imaging	as above
Hospital contacts regarding infection (any)	Records for all admissions or ambulatory contacts in North Denmark Region were reviewed. If an infection was noted (regardless of location), data was extracted
Microbiology	An extract for all received samples (regardless of anatomic site) was made from the database of the Department of Clinical Microbiology in the timespan from primary arthroplasty to study end
